# NSD1 gene evolves under episodic selection within primates and mutations of specific exons in humans cause Sotos syndrome

**DOI:** 10.1186/s12864-022-09071-w

**Published:** 2022-12-22

**Authors:** Vanessa I. Romero, Benjamin Arias-Almeida, Stefanie A. Aguiar

**Affiliations:** School of Medicine, Universidad San Francisco de Quito, Quito, Ecuador

**Keywords:** NSD1, Sotos, Macrocephaly, Selection, Episodic, primates

## Abstract

**Background:**

Modern human brains and skull shapes differ from other hominids. Brain growth disorders as micro- (ASPM, MCPH1) and macrocephaly (NFIX, GLI3) have been highlighted as relevant for the evolution in humans due to the impact in early brain development. Genes associated with macrocephaly have been reported to cause this change, for example NSD1 which causes Sotos syndrome.

**Results:**

In this study we performed a systematic literature review, located the reported variants associated to Sotos syndrome along the gene domains, compared the sequences with close primates, calculated their similarity, Ka/Ks ratios, nucleotide diversity and selection, and analyzed the sequence and structural conservation with distant primates. We aimed to understand if NSD1 in humans differs from other primates since the evolution of NSD1 has not been analyzed in primates, nor if the localization of the mutations is limited to humans. Our study found that most variations causing Sotos syndrome are in exon 19, 22 and 10. In the primate comparison we did not detect Ka/Ks ratios > 1, but a high nucleotide diversity with non-synonymous variations in exons 10, 5, 9, 11 and 23, and sites under episodic selection in exon 5 and 23, and human, macaque/colobus/tarsier/galago and tarsier/lemur/colobus. Most of the domains are conserved in distant primates with a particular progressive development from a simple PWWP1 in O. garnetti to a complex structure in Human.

**Conclusion:**

NSD1 is a chromatin modifier that suggests that the selection could influence brain development during modern human evolution and is not present in other primates; however, nowadays the nucleotide diversity is associated with Sotos syndrome.

**Supplementary Information:**

The online version contains supplementary material available at 10.1186/s12864-022-09071-w.

## Background

The brain of modern humans results from a unique neuronal growth pattern giving rise to a distinctive skull shape different from other archaic humans like Neanderthal and Denisovans [1]. Brain growth disorders as micro- and macrocephaly have been highlighted as relevant for the brain evolution in humans due to the impact in early brain development [2]. Mutations in microcephaly genes like assembly factor for spindle microtubules (ASPM), CDK5 regulatory subunit associated protein 2 (CDK5RAP2), microcephalin 1 (MCPH1), and centromere protein J (CENPJ) underwent pervasive positive selection during human evolution [3]. Macrocephaly disorders also provide insight in the context of brain growth by modifying the neuronal growth trajectory. For example, kinetochore scaffold 1 (KNL1 previously known as CASC5), is associated with gray matter volume difference. Mutations in phosphatase and tensin homolog (PTEN) in humans increase the lengthening of the prometaphase-metaphase not seen in chimpanzees [1]. Genes associated to macrocephaly like NFIX and GLI3 have been claimed to have played a role in the shaping of the human head and show numerous single nucleotide changes in non-coding regions [4, 5]. Another gene associated with macrocephaly is the nuclear receptor binding SET domain protein 1 or NSD1, from which mutations result in an overgrowth condition known as Sotos Syndrome [6, 7]. Here, we perform a systematic literature review of the condition, compare the nucleotide sequences within close primates, identify sites, and branches under selection, and describe the domain conservation among distant primates. Our findings conclude that the human branch evolves by episodic selection allowing mutations that result in Sotos syndrome.

Sotos syndrome is a rare genetic disorder characterized by overgrowth before and after birth [6, 7]. The disease has an incidence of 1/14000 and a prevalence of 1–9/10000 worldwide [8]. This syndrome is represented by facial features including long and narrow face, prominent forehead, flushed cheeks, and small pointed chin, macrocephaly, gigantism, advanced bone age, mild cognitive impairment, delayed motor, cognitive and social development, and speech impairments [9]. Less common clinical characteristics are seizures, scoliosis, heart and kidney defects, vision problems, and awkward gait. The abnormal growth resolves with time and the developmental delay may improve in the school-age years. The coordination problems persist during adulthood [6, 7].

NSD1 functions as a transcriptional intermediary factor capable of both negative and positive responses [9]. The gene controls normal growth and development, and mutations have been associated with overgrowth syndromes [10]. NSD1 has 3 isoforms, resulting from alternative splicing that target multiple receptors, such as androgen, estrogen, and retinoic acid. The important function of NSD1 suggests a constraint selection, however, the variety of disease phenotypes in humans demonstrate the opposite [11]. NSD1 is expressed in different primates such as chimpanzee, orangutan, and macaque. Visser et al. reported a similarity of 96.5–98.5% among a 3Kb region including NSD1 between chimpanzee and human [12]. Similar overgrowth presentations like Sotos syndrome have not been reported in primates. Therefore, we investigate the location of the mutations along the gene in humans, the nucleotide variation within primates and the conservation of sequence and structure of NSD1 among distant primates.

Our study analyzed all mutations reported in NSD1 for Sotos syndrome and localized them along on exons and functional domains. We compare the localization of the variations to sites under episodic selection in primates and support the association of macrocephaly causing genes to the modern head development of humans during evolution and not in primates or other archaic humans as Denisovans/Neanderthal. Finally, we describe the conservation of NSD1 among distant primates and identify a progressive development of the PWWP1 in upper clades of primates.

## Results

### Most human variations localized in exons 19, 22 and 20

**Fig. 1:**
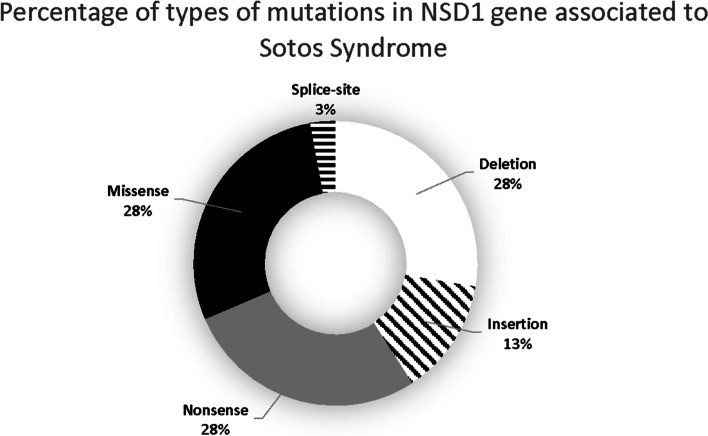
Percentage of types of mutations in NSD1 gene associated to Sotos Syndrome. Doughnut graph of the percentage of types of mutations in NSD1 gene associated to Sotos Syndrome. The most common are deletion, missense and nonsense followed by insertion and splice-site. White = deletion. Diagonal lines = insertion. Grey = nonsense. Black = missense. Straight lines = splice-sites

**Fig. 2:**
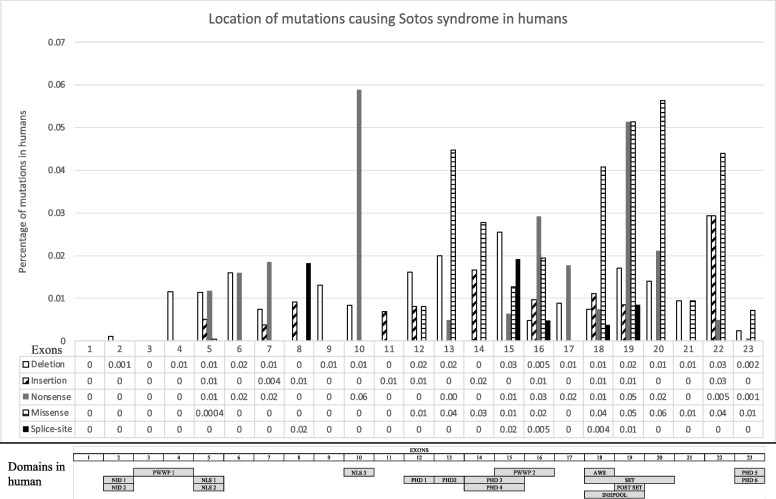
Location of mutations causing Sotos syndrome in human and the exon, and localization of the functional domains described in humans [10]. Total number of variants are 257. Upper graph: Y-axis is the percentage of mutations reported in humans. X-axis are the 23 exons along NSD1. Most deletions and insertions locate in exon 22, nonsense in exon 10, missense in exon 20 and splice-site in exon 15. White = deletion. Diagonal lines = insertion. Grey = nonsense. Black = missense. Straight lines = splice-sites. Lower graph: Localization of the functional domains described in humans along the NSD1 exons. PWWP = proline–tryptophan–tryptophan–proline domains, PHD1–PHD5 = plant homeodomains. PHD6 = variant C5HC3 PHD finger 6. AWS = pre-SET domain. NID = nuclear receptor-interaction. NLS = nuclear localization signals

We analyzed 304 reported cases and identified that most mutations associated with Sotos syndrome are missense, nonsense, and deletions; meanwhile, fewer common variants are splice-site, exonic deletions and unknown (Fig. 1, Table 1). After normalizing the number of variations to the length of each exon, we locate the most common deletions in exon 22, followed by 15, 13, 19 and 12, insertions in exon 22, followed by 14, 18, 16 and 8, and nonsense in exon 10, followed by 19, 16, 20 and 7. Missense variants are found the most in exon 20, followed by 19, 13, 22, and 16, and splice-site in exon 15, 8 and 18. The type of variant does not dictate the severity nor risk of complications that are associated with the syndrome. Within the NSD1 gene, exon 19 has most of the overall variations associated with Sotos syndrome, followed by exon 22, 20, 18 and 13 (Fig. 2, Table 1, Supplementary Table 2).

**Table 1:**
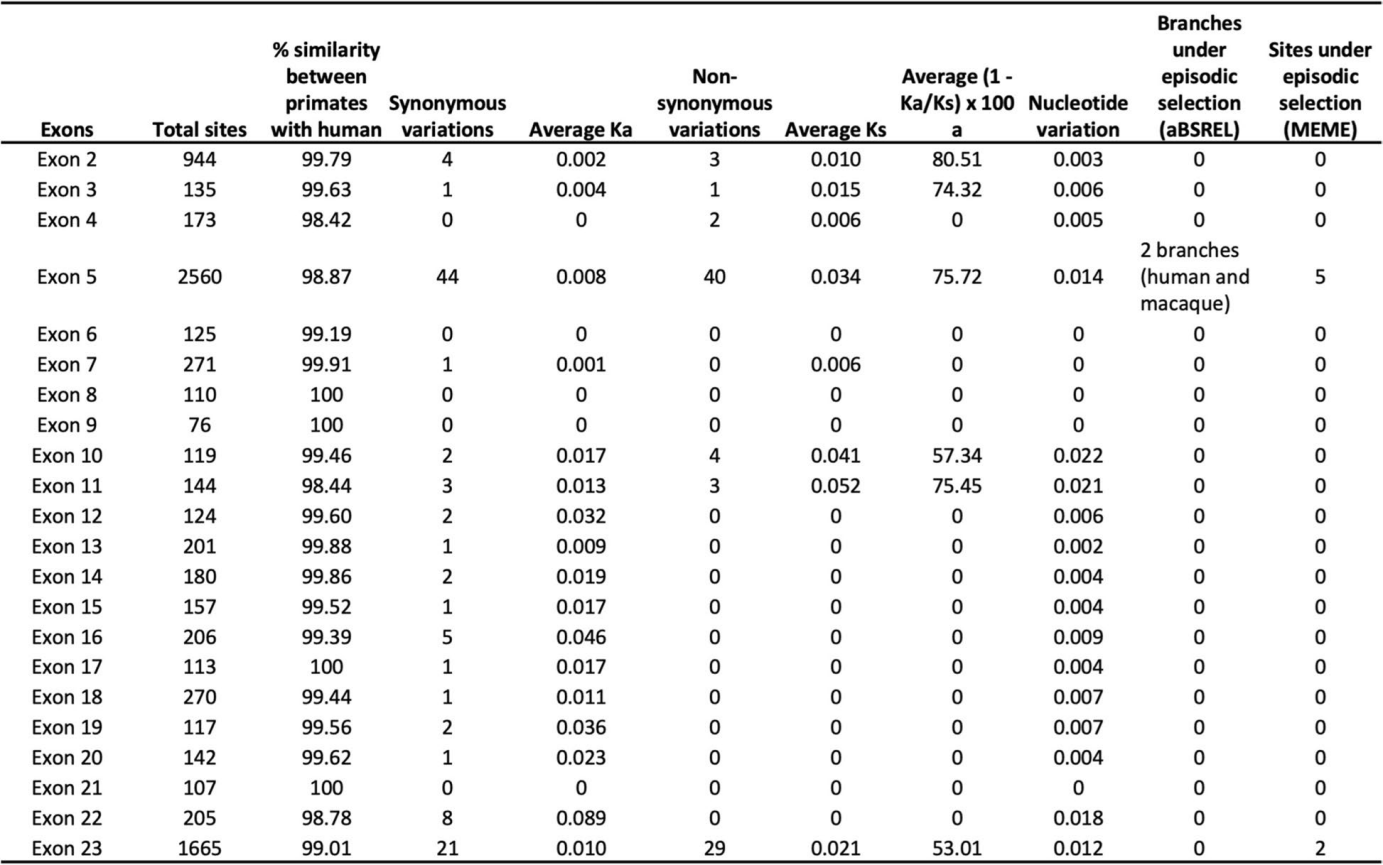
Comparison of each exon of NSD1 in chimpanzee, gorilla, orangutan, and macaque, with human

### High similarity between human and primates

Most of the human mutations are located in exon 19, 22 and 20, we tested whether these exons are conserved in primates or if the variability is limited to humans. The sequences from capuchin, galago and tarsier had undetermined regions and we could not identify for capuchin exon 23, galago exon 2–4, and for tarsier exon 2 and 18. All other primates share the number of exons and a similarity between 84 to 100%. Human, chimpanzee, and gorilla conserve 100% of their nucleotide sequences, except for exon 5, 11, 15, 18 and 23 (Table 1). Similar overgrowth presentations like Sotos syndrome have not been reported in primates. We performed alignment of all primates and none of the mutations that are pathogenic in humans are found in primates, suggesting that the nucleotide sequences mutated in the human patients are evolutionarily conserved among primates (Supplementary Table 6).

### Most substitutions between primates occur in exon 10, 5, 9, 11 and 23 and high nucleotide diversity between primates

Exons between species have a high similarity, therefore we questioned if the variants are synonymous or non-synonymous and which is the nucleotide diversity. We calculated whether the differences between species were either synonymous or non-synonymous and identified, after normalization, that most non-synonymous variations in exon 10, 5, 9, 11 and 23 (Fig. 2, Table 1). Under purifying selection, the number of nonsynonymous variations in a gene is expected to be smaller than the number of synonymous variations. Purifying selection can be measured by making pairwise comparisons ((1 − Ka/Ks) × 100), which indicate the percentage of synonymous variations [13]. We found that the average percentage of synonymous substitutions for exon 10, 5, 9, 11 and 23 was 71,71, 73.43, 74, 80.02 and 75.14% (Table 1). None of the pairs had Ka/Ks > 1 (Supplementary Table 3).

We calculated the total number of sites showing variation and the nucleotide diversity (π) between exons using the DNAsp6 program, as described in the Methods section. Exon 10 had high nucleotide variation with non-synonymous variations followed by exon 5, 9, 11 and 23 (Table 1). We then compared the π of NSD1 with that of conserved, rDNA, and diverse, polyubiquitin, genes. The π of NSD1 was comparable with that of polyubiquitin genes (range = 0.088 to 0.197) and much larger than those of rDNA genes (range = 0.00001 to 0.00018). This suggests that exon 10, 5, 9, 11 and 23 have values more similar to diversifying selection [14–16].

### Selective pressure in sites of exon 5 and 23, and human and macaque branches

We identified no diversifying selection per exon and examined selective pressure in sites and branches. We tested for selective pressure using one method for sites, MEME, and one for branches, aBSREL. We identified evidence of episodic or diversifying selection at 32 sites (167, 250, 317, 339 and 388) in exon 5, 17 sites in exon 23, 2 sites in exon 10 and 1 site in exon 6 using MEME. The branch analysis for diversifying selection detected higher ratio of nonsynonymous to synonymous substitution in human, galago, tarsier, macaque/colobus, macaque/colobus/tarsier and macaque/colobus/tarsier/galago from exon 5 and in tarsier and tarsier/lemur/colobus in exon 23 (Table 1 and Supplementary Table 4, Supplementary Table 5).

### Protein structure analysis among other species

**Fig. 3:**
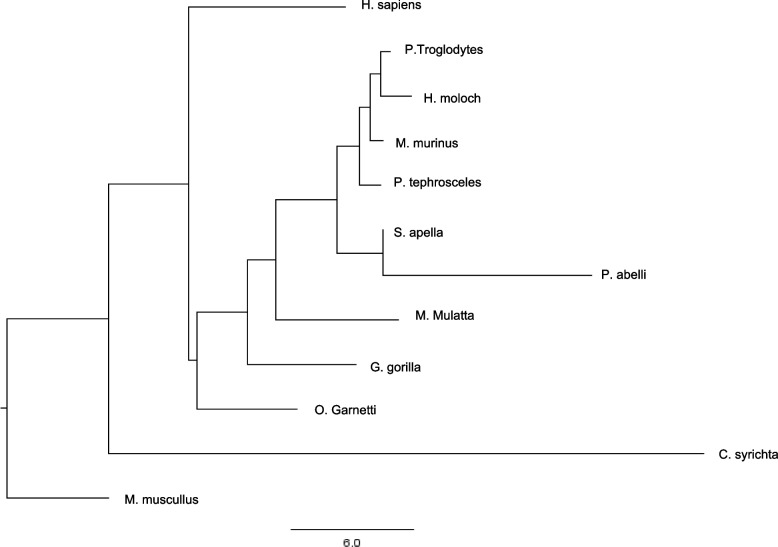
Dendrogram of the global differences among primate NSD1 protein structures. Structural differences considering the total structure of all species. Once a differential matrix is obtained structures are joined clustering the most resemble. The dendrogram does not follow the conventional primate phylogeny

**Fig. 4:**
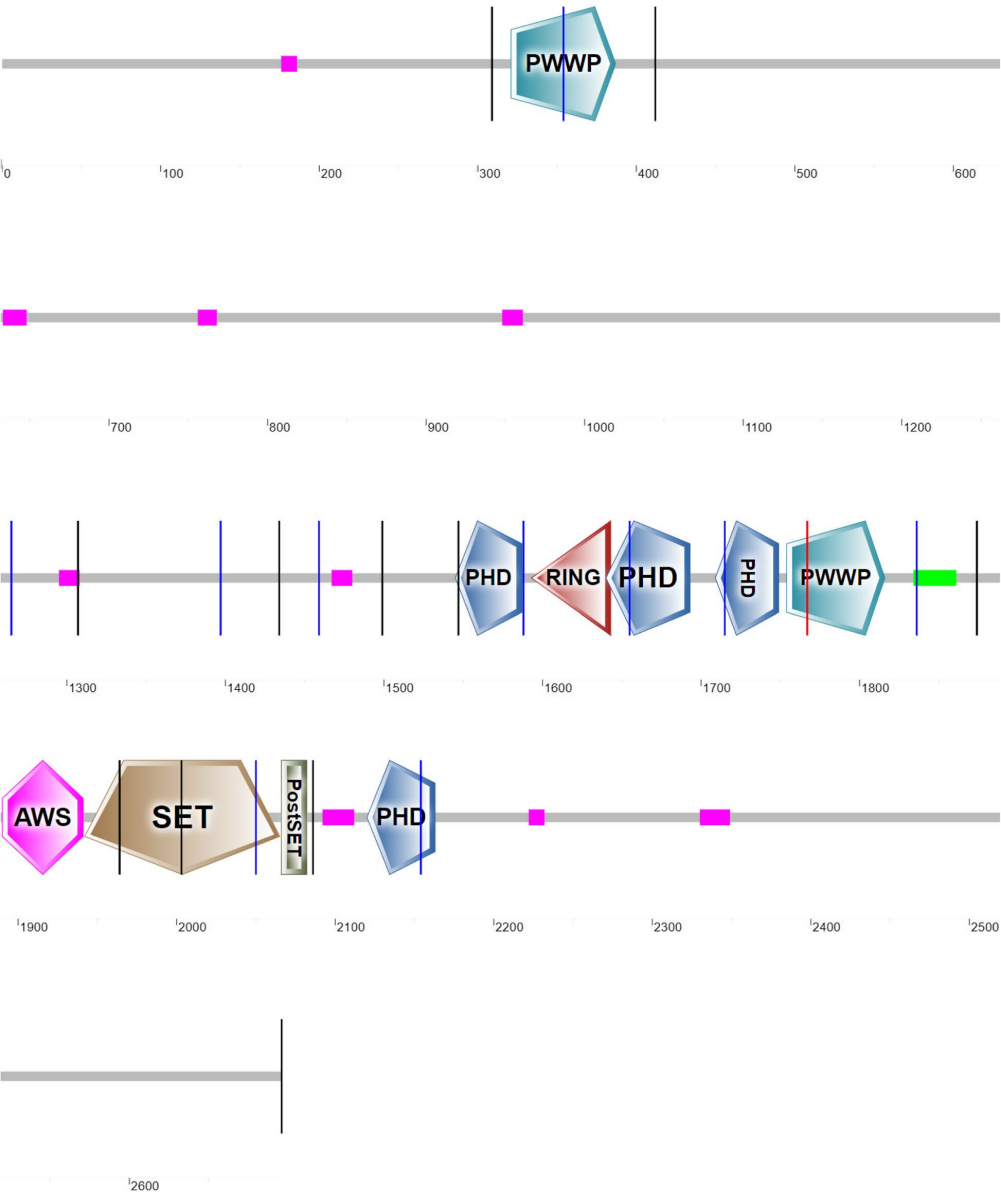
Schematic representation of human NSD1 protein domain organization. Vertical lines represent the site of intron splicing. From left to right: PWWP, PHD1, PHD6 = variant C5HC3 PHD finger 6. AWS = pre-SET domain. NID = nuclear receptor-interaction. NLS = nuclear localization signals. Most of the deletions, insertions, and nonsense variations occur in the NLS1 domain, missense in the PHD 5 and 6 domain and splice-site in the PWWP 2, PHD 3 and 4 domain. SMART predicted functional and catalytic domains, vertical lines represent sites of introns, pink rectangles represent low complexity regions and green rectangle coiled coil region. Simple predicted architecture is formed by 10 functional domains, PWWP 1–2, PHD 1–4, RING, AWS, SET and Post-SET

After analyzing the difference between close primates, we performed a comparison with distant primates as mentioned in Methods. We performed an analysis of the complete protein structure. *C. syrichta* localized at the base of the dendrogram and above of the out group suggesting an ancestral state. *Homo sapiens* grouped with the other primate species and does not follow the conventional primate phylogeny (Fig. 3, Supplementary Fig. 2, Supplementary Fig. 3) Therefore, we used the SMART software to identify the catalytic domains and divided the complete protein structure into two super domains as the differences between smaller regions could explain evolutionary divergence rather than the whole structure (Fig. 4). Superdomain 1 (SD1) (SD1:321–395) includes PWWP1 and Superdomain 2 (SD2) contains PWWP2, PHD 1–4, INHLOOP, AWS, SET and Post-SET domains (Supplementary Fig. 4, Supplementary Fig. 5, Supplementary Fig. 6, Supplementary Fig. 7, Supplementary Fig. 8, Supplementary Fig. 9, Supplementary Fig. 10, Supplementary Fig. 11, Supplementary Fig. 12, Supplementary Fig. 13, Supplementary Fig. 14).

**Fig. 5:**
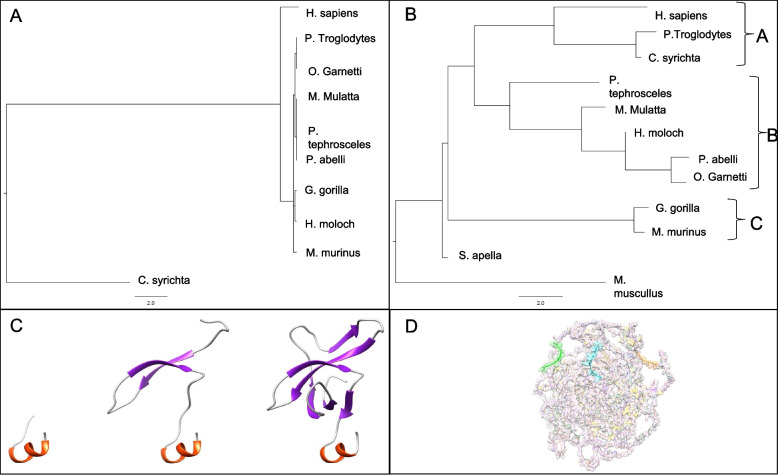
Structural relationships of SD1 and SD2 among primates, PWWP1 and NSL modelling A) SD1 structural relationships among primates. Super domain 1 is formed by the PWWP1 domain. *M. musculus* lacks the complete region and was excluded from the analysis. *Homo sapiens* separates from the rest of the primates, suggest a unique structure. B) SD2 structural relationships among analyzed species. Super domain 2 is formed by their central catalytic core of the protein, the SET domain. *S. apella* localized above of the outgroup. The rest of the species grouped in 3 clades. Clade A included *H. sapiens*, *P. troglodytes* and *C. syrichta*. Clade B grouped *P. tephrosceles*, *M. mulatta*, *H. Moloch*, *P. abeli* and *O. garnettii* in an ascending formation. Clade C included *G. gorilla* and *M. murinus*. C) The PWWP1 structure is composed of a five stranded beta barrel followed by a helix bundle. Left: *O. garnettii*, center: *C. syrichta*, right: human. The progressive changes between species suggest a complex evolution. D) NSLs distribution on NSD1 surface. Green: NSL1, cyan: NSL2 and orange NSL3. The three NSL have a triangular distribution around the surface allowing them to interact with other proteins

The complete sequence of SD1 was found in all species except for O. garnetti and *C. syrichta*. O. garnetti does not have the 5-beta strand topology that characterize the PWWP domains and *C. syrichta* has 2 of the 5 beta strand conformation of PWWP domains [17] (Fig. 5). We modelled the PWWP1 domain of O. garnetti, *C. syrichta* and *H. sapiens* and identified a simple structure in O. garnetti and a complex structure in *H. sapiens*, demonstrating the progressive development of the protein (Fig. 5, Supplementary Fig. 14). SD1 phylogenetic tree outgrouped *C. syrichta* at the base of the dendrogram and separated *Homo sapiens* from the rest of the primate species (Fig. 5). *M. musculus* lacks SD1 and was excluded.

We identified SD2 structures in all species, however O. garnetti lacks ~ 580 amino acids in the C-termini which includes the last PHD zinc finger of the second super domain (SD2:1545–2206) (Fig. 5). All other primate species PHD 1–4, PWWP2, AWS, and INHLOOP domains, were highly conserved in sequence and structure (Fig. 5, Table 2, Supplementary Fig. 14, Supplementary Fig. 15). In the SD2 phylogenetic analysis, *S. apella* localized at the base of the dendrogram and above of the outgroup. The rest of the species are grouped in 3 clades names as A-C. Clade A included *H. sapiens*, *P. troglodytes* and *C. syrichta*. Clade B grouped *P. tephrosceles*, *M. mulatta*, *H. Moloch*, *P. abeli* and *O. garnettii* in an ascending formation. Clade C included *G. gorilla* and *M. murinus* (Fig. 5, Table 2). O. garnetti lacks a complete SD2 and was excluded. SET and Post-SET domain sequences vary outside the functional amino acids in orangutan and tarsius.

**Table 2:** Structure and sequence variation of species that present variations in functional domains of NSD1

Domain	Function	Structure	Sequence
PWWP	Present in DNA binding proteins, and involve on protein-protein interactions	deletion: O. garnetti, *C. syrichta*	deletion: O. garnetti, *C. syrichta*
NLS1	Translocation signal sequence involve in protein localization inside nucleous	CONSERVED	SNPs: *M. musculus*, *C. syrichta*, SNPs: *S. apella*, O.garnettii
NLS2		CONSERVED	SNPs: *M. murinus*
NLS3		CONSERVED	SNPs: *C. syrichta*, *M. musculus*, SNPs: O.garnettii
PHD1	C4HC3 zinc-finger-like motif found in nuclear proteins involved in epigenetics and chromatin regulation	CONSERVED	SNPs: muscullus, *S. apella*
PHD2		CONSERVED	SNPs: *M. murinus*, M. muscullus, P. thephrosceles
RING	E3 ubiquitin-protein ligase activity, binding activity towards E2 ubiquitin-conjugating enzymes	CONSERVED	SNPs: M. muscullus, *M. murinus*, O.garnettii, P. thephrosceles
PHD3	C4HC3 zinc-finger-like motif found in nuclear proteins involved in epigenetics and chromatin regulation	CONSERVED	SNPs: *C. syrichta*, *M. murinus*, O.garnettii
PHD4		CONSERVED	CONSERVED
PWWP2	Present in DNA binding proteins, and involve on protein-protein interactions	CONSERVED	CONSERVED
AWS	Associated With SET, suggesting a role in gene regulation by methylation of lysine residues in histones and other proteins.	CONSERVED	CONSERVED
SET	Histone methyltransferase, modulate gene activities and/or chromatin structure, necessary for the methylation of lysine-9 in the histone H3 N terminus	CONSERVED	SNPs: P. abelli
INHLOOP	Inhibition loop for maintenance of inactive state.	CONSERVED	CONSERVED
Post-SET	Cysteine-rich regions, play a crucial role when it comes to substrate recognition and enzymatic activity	CONSERVED	SNPs: *C. syrichta*
PHD5	C4HC3 zinc-finger-like motif found in nuclear proteins involved in epigenetics and chromatin regulation	Present different conformation: *M. musculus*	Deletion: O. garneti

Other regions not included in the superdomains (NLS1, NLS2, NLS3) were highly conserved (Table 2). We modelled the human sequence of NLS 1–3 domains and located them at the external region of the protein on a triangular orientation to recognize other proteins (Fig. 5).

## Discussion

Genes associated with macrocephaly have been claimed to play a role in shaping the modern human head [4, 5]. In humans, several amino acid substitutions, nonsense, and frameshift, of NSD1 cause an overgrowth and macrocephaly known as Sotos syndrome. First, we locate that with variations in exon 19 (SET and Post-SET domains), 22 and 20 [6, 7] (Fig. 1). These exons do not have nonsynonymous variations or disorders like Sotos syndrome in other primates and we suggest a selection in modern humans. Second, we investigated NSD1 selection comparing the human NSD1 with close and distant primates by detecting percentage of similarity, Ka/Ks ratios, nucleotide diversity and signatures of episodic selection. We found no Ka/Ks ratios (> 1) in our within-species comparisons but a high nucleotide diversity and detected selected sites of episodic selection in exon 5, 23, 10 and 6, and in human, galago, tarsier, macaque/colobus, macaque/colobus/tarsier and macaque/colobus/tarsier/galago and tarsier and tarsier/lemur/colobus branches. Finally, we categorized the sequence and structure conservation of NSD1 domains among distant primates into three groups and identified a progressive development in PWWP1.

### NSD1 mutations in human and functional domains

NSD1 has 23 exons, three isoforms with 16 domains, and codes for H3K36me2 protein (Fig. 2). H3K36me2 recruits DNMT3A allowing the maintenance of DNA methylation in intergenic regions by colocalizing at noncoding regions of euchromatin. NSD1 gene is expressed in the brain, kidney, skeletal muscle, thymus, and peripheral blood leukocytes. 90 percent of Sotos syndrome are caused by NSD1 mutations. The most common types of mutation include missense, nonsense and frameshift deletions resulting in overgrowth with distinctive facial characteristics. In humans, most variations are located from exon 10 to 22 which are NLS3, PHD2, PHD3, PHD4, PWWP2, AWS, SET, and Post SET domains (Table 1, Fig. 1, Fig. 2). PHD and SET function in histone methylation and as ubiquitin E3 ligases. NLS domains play essential roles for the translocation and PWWP region is involved in DNA methylation, DNA repair and regulation of transcription [10]. Similarly, to NSD1, ASPM (abnormal spindle-like, microcephaly-associated; MCPH5) undergoes positive selection in selected exons throughout the primate lineage leading to humans and currently is associated to microcephaly [18]. Microcephalin (MCPH1), shows a strong signature of positive selection in specific exons primarily in the lineage leading from the ancestral primates to the great apes [19]. Both CDK5RAP2 (CDK5 regulatory-subunit-associated protein 2; MCPH3) and CENPJ (centromeric protein J; MCPH6) report higher rates of non-synonymous substitutions in primates than rodents, and CDK5RAP2 shows especially high rates in the human and chimpanzee terminal lineages [20].

### Methyltransferases vary in evolution

One function of NSD1 includes the upstream binding to the bone morphogenetic protein 4 promoter, which increases H3K36 methylation and promotes bone morphogenetic protein 4 transcription [21]. NSD1 is a methyltransferase which regulates the epigenome during development, and we suggest it was implicated in the development of the human brain. We demonstrated the similarity between primates to be high due to the histone methyltransferases activity and consider that variations alter the functionality (Table 1). The high nucleotide diversity between primate’s exons is comparable with that of diversifying genes like polyubiquitin. Exon 10 (NLS3), 5, 9, 11 and 23 had more non-synonymous than synonymous between species. Mutations in exons 10 and 5 are detected in Sotos syndrome, however there are not many reported in exons 11, 9 or 23 (Fig. 2). We observed more variants in exon 22 associated with Sotos than exon 23. Exon 23 includes PHD5 and PHD6 which might be necessary for the proper function of NSD1, and the lack of this exon due to variants in exon 22 is pathogenic. Variants in exon 23 could retain functionality of PHD5 and PHD6.

NSD1 is a member of the NSD family of SET domain-containing histone methyltransferases (NSD1, NSD2 and NSD3). The NSD family have specific mono- and demethylase activities for H3K36, carry nonredundant roles during development and aberrant expression is associated with multiple diseases [22]. During mouse development, NSD1 is expressed in the telencephalic region of the brain and spinal cord [23]. After birth, NSD1 expression is predominantly neuronal within the cerebral cortex and in a smaller proportion in astrocytes and oligodendrocytes [24]. In humans, NSD1 variations are associated with overgrowth syndromes, with macrocephaly, and to the evolution of modern human brains and skull shape. NSD2 haploinsufficiency is associated with Wolf-Hirschhorn syndrome characterized by heart defects and severe mental and growth retardation [25]. SETD1A, a methyltransferase like NSD1, indirectly regulates neurogenesis through WNT/β-CATENIN signal with variations limited to modern human and absent in Neanderthal/Denisovan [5]. Mixed lineage leukemia protein-1 (MLL1) a member of the SET1 family of H3K4 methyltransferases highly conserved from yeast to humans. GLI3 and NFIX have been associated with evolution in the human lineage and currently to disease, and our project is the first to describe NSD1. Hypermethylation of NFIX in anatomically modern humans influenced the balance between lower and upper projection of the face compared to other species [26]. In humans, NFIX mutations are associated with impair speech capabilities, Marshall–Smith and Malan syndromes [26]. GLI3 regulators were found to show the signatures of positive selection, are unique to modern human lineage and possibly lead to the evolutionary human brain development. Selection may act to increase the frequency of de-novo beneficial mutations [27]. The phenotypic spectrum of GLI3 mutations includes autosomal dominant Greig cephalopolysyndactyly syndrome and Pallister–Hall syndrome [28].

Nucleotide variations in this complex have been reported to alter the function and are correlated with different evolutionary lineages [29]. We suggest that chromatin modifiers like NSD had relaxed selection towards brain development during modern human evolution, however nowadays the relaxed selection is associated with brain growth and facial disorders.

### NSD1 family related genes and other genes are associated to evolution and disease in modern humans

Episodic selection is a process in which codons experience purifying selection with bursts of strong positive selection within certain lineages. The specific codons experience positive selection, followed by purifying selection maintaining the variant and plays a role in in adaptive evolution [30]. We identified sites under episodic selection in exon 5 (NLS 1 and 2 domain) and exon 23 (PHD5 and 6 domain) that could modify the function as seen in other SET1 proteins (Fig. 3, Table 1). We detected episodic selection in human and macaque branches and support the evidence of selection in humans and no other archaic hominid. Modern human brains and skull shape differ from other hominids as a result of nucleotide variations in regulatory regions during early cortical development [4, 5]. CASC5, required for the kinetochore-microtubule attachment, is associated with higher gray matter volume [31]. Higher expression of PTEN elevates Beta-Catenin signaling controls the correct neuron positioning, dendritic development, and synapse formation [32]. The transcription factor TCF3 represses Wnt-Beta-Catenin signaling and neuronal differentiation, increasing the neural stem cell population during neocortical development [33]. NFIX1 and NSD1 genes are associated with macrocephaly and Sotos syndrome and were described to be important in the shaping of the modern human head [34, 35]. Variation also had side effects such as neurodevelopmental disorders affecting brain growth and facial features [4]. Various genes enriched in modern humans are disease-relevant genes like CHD8 and CPEB4 in autism spectrum, HTT in Huntington’s disease, FOXP2 in language impairment [5]. Likewise, NSD1 is associated with Sotos and Weaver syndrome and only in specific exons. We did not infer the divergence time of NSD1 variation in primates however there is evidence of episodic selection during human brain evolution. Humans have heavier brains compared to other primates (1400 g vs 395-490 g) [36]. Human brain mass increased during the divergence of Australopithecines [37]. The neocortex enlarged in the archaic hominin lineage after the divergence of chimpanzees (6–7 million years ago). The cranial lobe size differs between anatomically modern humans and Neanderthals, which indicates unique neocortical regions evolving in humans [38]. We obtained the frequency of NSD1 variants in the human populations from The Genome Aggregation Database (gnomAD) to identify whether a specific group within humans are subject to selection. Nine variants had a frequency higher than 0.05 (5 synonymous, 2 located in introns and 2 missense) were classified as benign and not specific to a population (Supplementary Table 7).

### Primate protein structure analysis prediction and evolutionary relations

Whole protein dendrogram demonstrated that the tarsius *C. syrichta* and the mouse are closely related, however the rest of the tree did not follow the conventional primate phylogeny and we divided the sequence into superdomains. (Fig. 3). Most of the species have a similar SD1 and SD2 structure, except for O. garnetti and *C. syrichta* (Fig. 5, Table 2). SD1 and SD2 phylogenetic analysis confirmed that humans diverged from the rest of non-hominoid primates. SD1 analysis outgrouped *Homo sapiens* from other primates suggesting a uniqueness in this species. SD1 consists of the PWWP domain named after the central core Pro-Trp-Trp-Pro, which functions as a transcription factor [39, 40]. SD2 contains PWWP2, PHD 1–4, AWS, SET and Post-SET domains. We identified that the functional domains inside the structure (PHD 1–4, PWWP2, AWS, and INHLOOP domains) that mainly act as nuclear signaling, DNA binding and interactions with other proteins are highly conserved. The plant homeodomain (PHD) is a zinc finger motif found in nuclear proteins involved in epigenetics and chromatin regulated transcription. PHD functions as a protein-protein interaction domain, and cooperates with BROMO domains for nucleosome binding in vivo [41–43]. The inhibitory loop (INHLOOP) is an amino acid sequence found between SET and Post-SET, normally inhibiting NSD1 function and is associated with abnormal expression of genes in cancer [44, 45]. AWS and SET domain interaction regulates gene expression by methylation lysine in proteins like histones [46]. SET and Post-SET domain sequences vary outside the functional amino acids in orangutan and tarsius. SET structure includes turns and loops and Post-SET is a cysteine rich sequence necessary for the SET domain catalysis [47–49]. Other regions not included in the superdomains (NLS1, NLS2, NLS3) were highly conserved (Fig. 5, Table 2). NLS functions as a nuclear signal [10]. We found that exon 10 has the highest non-synonymous variants, and the highest number of nonsense variants in humans. Exons 9 and 11 do not have a functional domain therefore variation is allowed in primates and is not as pathogenic in humans as other mutations. Episodic selection occurs preferably in exon 5 (NLS1 and NLS2) and 23 (PHD5 and PHD6) which are the longest exons. Most variants in humans are located in exon 5 and 23, however after normalizing the number of variations to the length of the exon the most common variants were in exon 19, 22, 20 and 18 (AWS, SET and Post-SET). One scenario could be that in other primates NLS - a nuclear domain- function is allowed to differ more than the SET domains. In contrast, human SET domains vary resulting in brain change. Another scenario could be that NLS variants in humans are damaging and individuals die in utero, therefore the frequency is underrepresented.

We analyzed the differences in structure and amino acid sequence and categorized three groups. The first group has a highly conserved sequence and structure, which are PDH4, PWWP2, AWS, and INHLOOP domains (Table 2, Fig. 5). The second group has a conserved structure but differs in aminoacid sequence and includes NSL1, NSL2, NSL3, PHD1, PHD2, PHD3, SET and Post-SET domains (Table 2, Fig. 5). The third includes PWWP1 and PHD5 domains, and are the least conserved in sequence and structure, especially for the prosimian species O. garnetti and *C. syrichta* (Table 2, Fig. 5). We suggest that amino acids change by exposure to biochemical niches in regions between species depending on their gene expression, cellular activity, and quantum dynamics. The developmental progression of PWWP1 from O. garnetti, *C. syrichta* to *H. sapiens* and the lack of PHD5 in O. garnetti, suggest a novel function in the upper clades of primates. The conserved regions must remain unchanged due to their important function like the catalytic performance and molecular signaling involved in chromatin remodeling, signaling, and protein interaction [22, 50]. The PWWP1 domain is found in transcription factors or proteins involved with nuclear regulation. This domain is involved in protein-protein interaction, DNA binding/recognition, controls the function of NSD1 and plays a central role on cellular growth and differentiation of neural crest cells. Modifications in PWWP alters the regulation and specialization of various nuclear processes. The progressive changes in this domain describe the molecular specialization of the chromatin regulation process during primates’ evolution [39, 40].

## Conclusion

We conclude that chromatin modifiers like NSD1 could influence brain development during human evolution and are not present in other primates, and nowadays the nucleotide diversity is associated with Sotos syndrome. Most of the mutations associated with Sotos syndrome are located in exon 19 which is the SET and Post-SET domains, and a high nucleotide diversity within primates occurs in exon 10 which is a NLS 3 domain. Human and macaque branches evolve under episodic selection, identified sequence and structural conservation among distant primates and describe a progressive development from a simple PWWP1 in O. garnetti to a complex structure in humans. We suggest that other macrocephaly genes could evolve under episodic selection in the human branch and differ in primates and other archaic humans as Neanderthals and Denisovans.

## Methods

### Systemic literary review of Sotos syndrome

We performed a systematic literature review (keywords: clinical trial, meta-analysis, and review Sotos syndrome or NSD1) in the databases PubMed, Scielo and Google scholar. We identified 304 reported variants between the years 2003–2021 and excluded the articles that included animal models, cancer, and alternative treatments for Sotos syndrome’s complications. PubMed database had 504 articles, Scielo 12 articles, and Google Scholar 10, 500 papers (Supplementary Fig. 1 and Supplementary Table 1).

### NSD1 sequences in primates

We obtained the DNA sequences of NSD1 from the National Center for Biotechnology Information (NCBI) gene database (http://www.ncbi.nlm.nih.gov/gene/) for the following primate species: *H. sapiens* (NC 000005.10), *P. troglodytes* (NC 036884.1), *G. gorilla* (NC 044607.1), *P. abeli*i (NC 036908.1), *M. mulatta* (NC 041759.1), *Hylobates Moloch* (NW 022611648.1), *Sapajus apella* (NW 022437140.1, *Carlito syrichta* (NW 007089627.1), *Microcebus murinus* (NC 033691.1), *Otolemur garnettii* (NW 003852656.1), and *Piliocolobus tephrosceles* (NC 045437.1). We reconstructed the 22 exons from primates using the sequences described by Kurotaki et al. [51]. Exon 1 was not reported; therefore, it was excluded.

### Multiple alignment, percent identity matrix and disparity index

We performed multiple alignment and Percent Identity Matrix of the primate DNA sequences using the online software Clustal omega [52]. Percent Identity Matrix calculates the percentage of similarity between two nucleotide sequences; the higher the number, the higher the similarity [53]. The disparity index was calculated by comparing the nucleotide frequencies in each pair of sequences, using the number of observed differences between sequences [54].

### Estimation of polymorphic/variant sites, nucleotide diversity, and ratio of synonymous and nonsynonymous sites

Ka/Ks is the ratio of the number of nonsynonymous nucleotide substitutions per total number of nonsynonymous sites for each codon (Ka), to the number of synonymous nucleotide substitutions per total number of synonymous sites for each codon (Ks) [55, 56]. We estimated the Ka/Ks ratio for each pair of exons using the program DNAsp6. The level of purifying selection was then calculated as (1 − Ka/Ks) × 100. Nucleotide diversity (π), the average number of nucleotide differences per site between sequences, was calculated using the DNA polymorphism option in DNAsp6 [56].

### Inferring selective pressure

We used two methods for inferring the strength of natural selection, Mixed Effects Model of Evolution (MEME) and adaptive Branch-Site Random Effects Likelihood (aBSREL).

MEME detects sites evolving under episodic selection under a proportion of branches by using a mixed-effects maximum likelihood approach for episodic diversifying selection [57] .

aBSREL models site-level and branch-level ω heterogeneity and tests for each whether a proportion of sites have evolved under episodic selection. After aBSREL fits the full adaptive model, the Likelihood Ratio Test is performed at each branch and compares the full model to a null model [58].

### Protein sequences phylogenetic analysis

NSD1 primate protein sequences where retrieve for NCBI data base, *Homo sapiens* (NP_071900.2), *Macaca mulatta* (XP_014996983.1), *Pan troglodytes* (XP_016809812.1), *Gorilla gorilla gorilla* (XP_018882446.1), *Pongo abelii* (XP_024102922.1), *Pan paniscus* (XP_003806901.1), *Hylobates Moloch* (XP_032014602.1), *Sapajus apella* (XP_032125809.1), *Carlito syrichta* (XP_008048621.1), *Microcebus murinus* (XP_012642771.1), *Otolemur garnettii* (XP_023364388.1), *Piliocolobus tephrosceles* (XP_023040770.1), *Mus musculus* (XP_006517209.1). *Homo sapiens* were used as template. Sequences were aligned using MUSCLE algorithm [59]. Two phylogenetic statistical analysis were performed using PhylML platform. Bayesian analysis was performed on Mr. Bayes using GTR as number of substitutions, mtREV+G as substitution model, for 10.000 generations, sampling each 100 trees and burning the 5% of the initial trees. For Maximum Likelihood analysis, Dayhoff was used as the substitution model for 500 generations [60]. *Mus musculus* was used as the outgroup. The resulting trees were drawn using Figtree software (http://tree.bio.ed.ac.uk/software/figtree/). Sequence alignments were visualized using Genius Prime Pro [61].

### Protein modeling analysis

*Homo sapiens* (AF-Q96L73-F1) predicted protein structure was retrieved from AlphaFold2 platform and used as template for the structural modeling prediction of primate homologs [62]. Models were obtained from SWISS-MODEL platform [63]. Protein structures were compared using all against all option and whole structure and super domains (SD) comparison included in the DALI software [64, 65]. We plotted all dendrograms using Figtree software. For a specific structure-functional analysis the two main catalytical domains were isolated and analyzed. *Homo sapiens* domains were characterized using SMART software [46]. For the creation of visual alignment of primates homologs structures and regions of interest with the *Homo sapiens* reference we used Chimera software [66].

## Supplementary Information

**Additional file 1:** **Supplementary Fig 1.** PRISMA of SLR of NSD1 mutations associated with Sotos syndrome in humans. **Supplementary Fig 2.** Maximum likelihood amino acid evolution tree. **Supplementary Fig 3.** Bayesian inference amino acid evolution tree. **Supplementary Fig 4.** PWWP1 domain protein structure alignment. **Supplementary Fig 5.** PHD5 domain protein structure alignment. **Supplementary Fig 6.** PHD1 domain protein structure alignment. **Supplementary Fig 7.** PHD2 domain protein structure alignment. **Supplementary Fig 8.** PHD3 domain protein structure alignment. **Supplementary Fig 9.** PHD4 domain protein structure alignment. **Supplementary Fig 10.** PWWP2 domain protein structure alignment. **Supplementary Fig 11.** AWS domain protein structure alignment. **Supplementary Fig 12.** SET domain protein structure alignment. **Supplementary Fig 13.** Post-SET domain protein structure alignment. **Supplementary Fig 14.** Localization of SD1 and SD2 on the modelled structure. **Supplementary Fig 15.** SD2 distribution of functional domains. Super domain 2 was divided in. two for a more comprehensive distribution analysis.

**Additional file 2:** **Supplementary Table 1.** Scientific paper, localization in the exon and type of variation. **Supplementary Table 2.** Percentage of similarity between nucleotide sequences of chimpanzee, gorilla, orangutan, and macaque and human. **Supplementary Table 3.** The Ka, Ks and (1 − Ka/Ks)   100 of NSD1 within five primate species. **Supplementary Table 4.** Sites yielded a statistically significant for episodic positive/diversifying selection. **Supplementary Table 5.** Total branches tested for diversifying selection. **Supplementary Table 6.** Alignment of nonsense and missense variants of human and primates. **Supplementary Table 7.** The Genome Aggregation Database frequency of variants by population.

## Data Availability

All data generated or analyzed during this study are included in this published article and its supplementary information files.

## Abbreviations

NSD1: nuclear receptor binding SET domain protein 1
Π: nucleotide diversity
PWWP: Pro-Trp-Trp-Pro
PHD: plant homeodomain
INHLOOP: inhibitory loop
