# Supplementary material for: NSD1 gene evolves under episodic selection within primates and mutations of specific exons in humans cause Sotos syndrome
Source: BMC Genomics. 2022 Dec 22;23:849. doi: 10.1186/s12864-022-09071-w (PMC9783842; doi:10.1186/s12864-022-09071-w)
Supplement: Supplementary file 1 — Additional file 1: Supplementary Fig 1. PRISMA of SLR of NSD1 mutations associated with Sotos syndrome in humans. Supplementary Fig 2. Maximum likelihood amino acid evolution tree. Supplementary Fig 3. Bayesian inference amino acid evolution tree. Supplementary Fig 4. PWWP1 domain protein structure alignment. Supplementary Fig 5. PHD5 domain protein structure alignment. Supplementary Fig 6. PHD1 domain protein structure alignment. Supplementary Fig 7. PHD2 domain protein structure alignment. Supplementary Fig 8. PHD3 domain protein structure alignment. Supplementary Fig 9. PHD4 domain protein structure alignment. Supplementary Fig 10. PWWP2 domain protein structure alignment. Supplementary Fig 11. AWS domain protein structure alignment. Supplementary Fig 12. SET domain protein structure alignment. Supplementary Fig 13. Post-SET domain protein structure alignment. Supplementary Fig 14. Localization of SD1 and SD2 on the modelled structure. Supplementary Fig 15. SD2 distribution of functional domains. Super domain 2 was divided in. two for a more comprehensive distribution analysis. [file 12864_2022_9071_MOESM1_ESM.pdf]

Supplementary Figures

Supplementary Fig. 1. PRISMA of SLR of NSD1 mutations associated with Sotos syndrome in humans.

Supplementary Fig. 2. Maximum likelihood amino acid evolution tree.

Supplementary Fig. 3. Bayesian inference amino acid evolution tree.

Supplementary Fig. 4. PWWP1 domain protein structure alignment.

Supplementary Fig. 5. PHD5 domain protein structure alignment.

Supplementary Fig. 6. PHD1 domain protein structure alignment.

Supplementary Fig. 7. PHD2 domain protein structure alignment.

Supplementary Fig. 8. PHD3 domain protein structure alignment.

Supplementary Fig. 9. PHD4 domain protein structure alignment.

Supplementary Fig. 10. PWWP2 domain protein structure alignment.

Supplementary Fig. 11. AWS domain protein structure alignment.

Supplementary Fig. 12. SET domain protein structure alignment.

Supplementary Fig. 13. Post-SET domain protein structure alignment.

Supplementary Fig. 14. Localization of SD1 and SD2 on the modelled structure.

Supplementary Fig. 15. SD2 distribution of functional domains. Super domain 2 was divided in two for a more comprehensive distribution analysis.

Supplementary figures

Supplementary figure 1. PRISMA of SLR of NSD1 mutations associated with Sotos syndrome in humans.

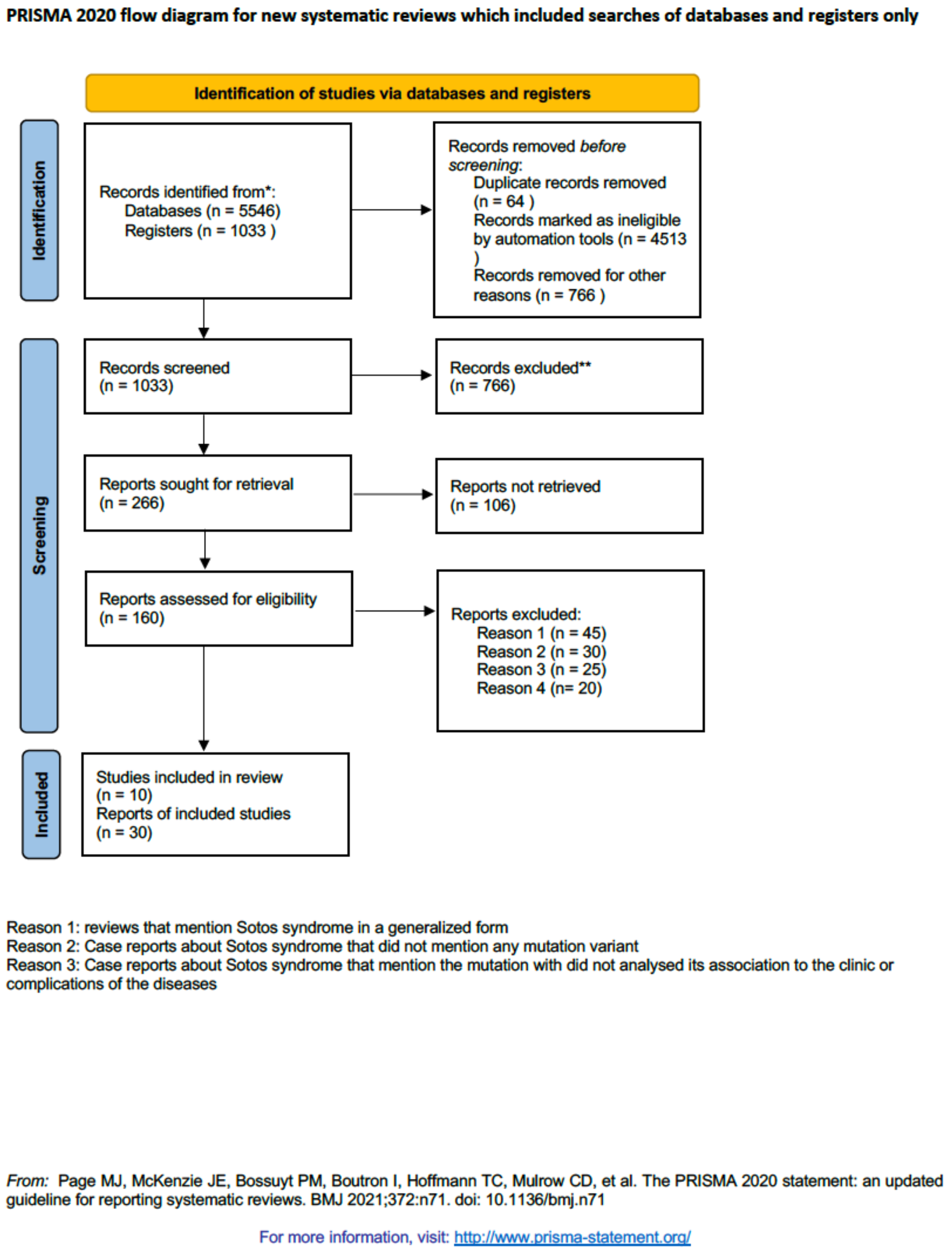

Supplementary figure 2. Maximum likelihood amino acid evolution tree.

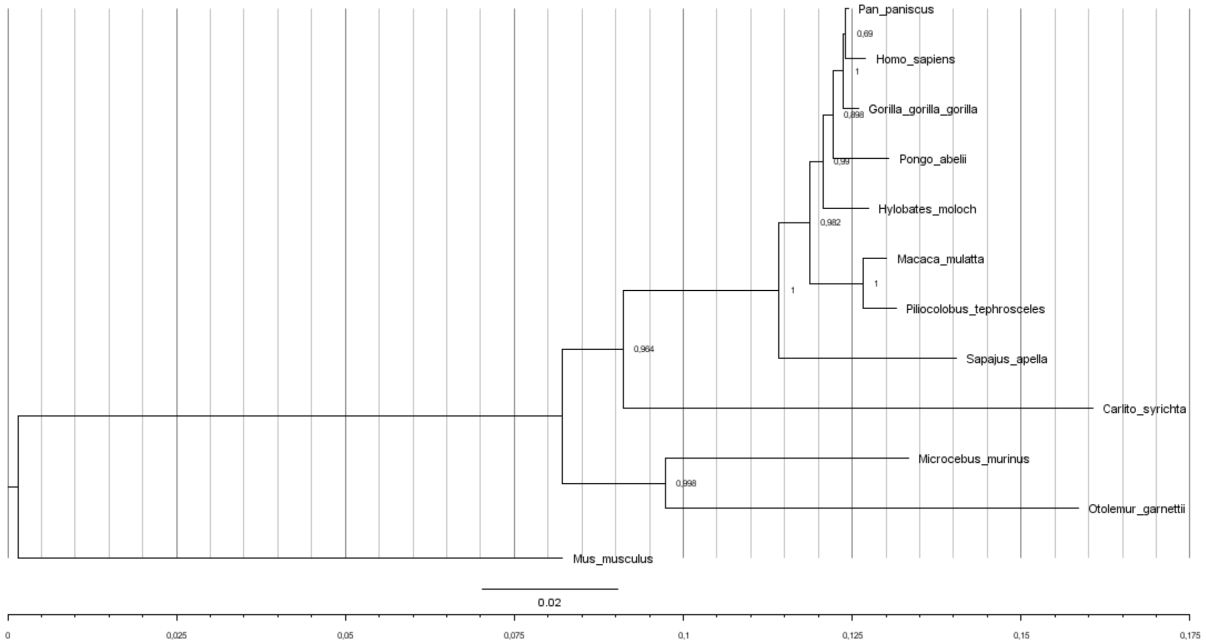

Supplementary figure 3. Bayesian inference amino acid evolution tree.

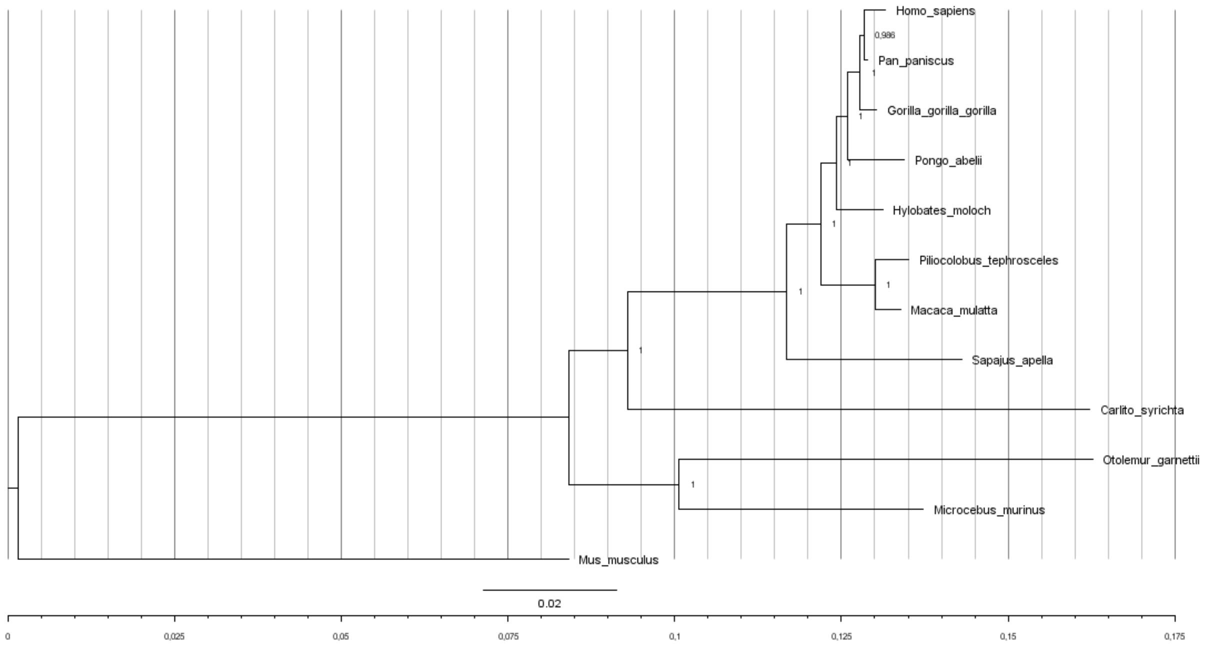

Supplementary figure 4. PWWP1 domain protein structure alignment.

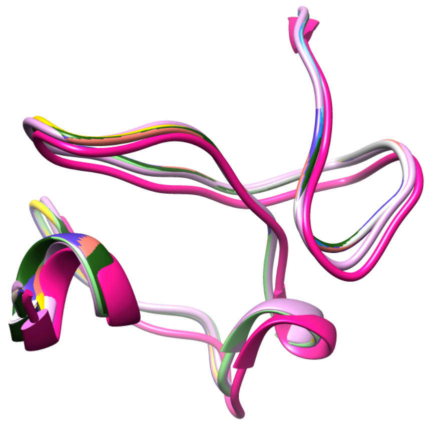

Supplementary figure 5. PHD5 domain protein structure alignment.

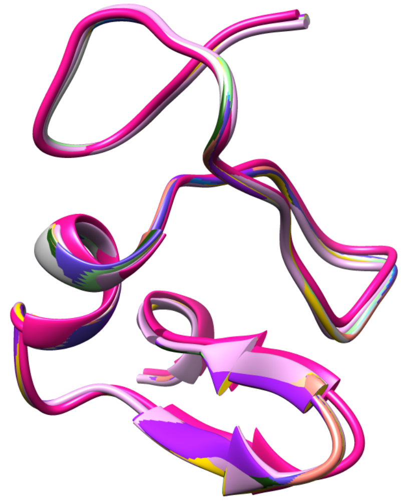

Supplementary figure 6. PHD1 domain protein structure alignment.

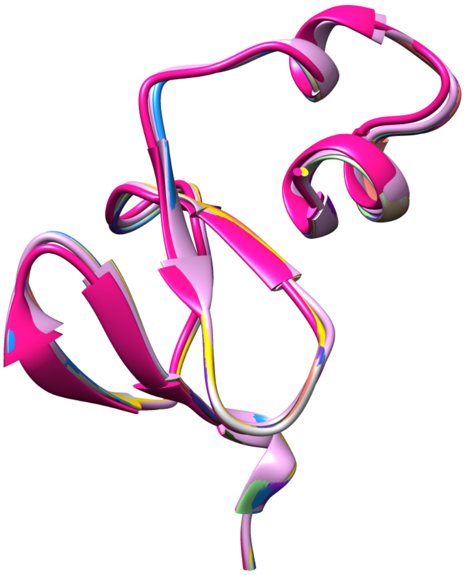

Supplementary figure 7. PHD2 domain protein structure alignment.

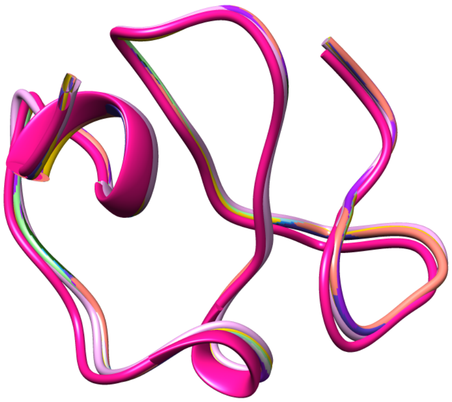

Supplementary figure 8. PHD3 domain protein structure alignment.

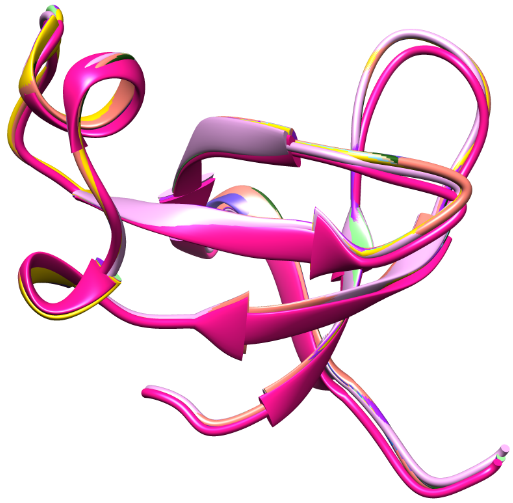

Supplementary figure 9. PHD4 domain protein structure alignment.

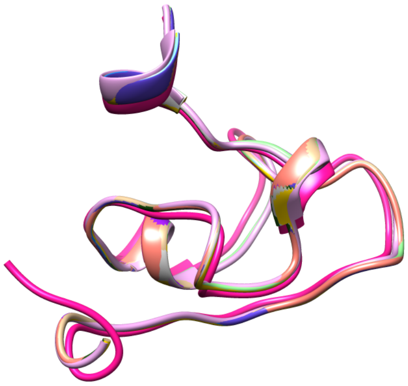

Supplementary figure 10. PWWP2 domain protein structure alignment.

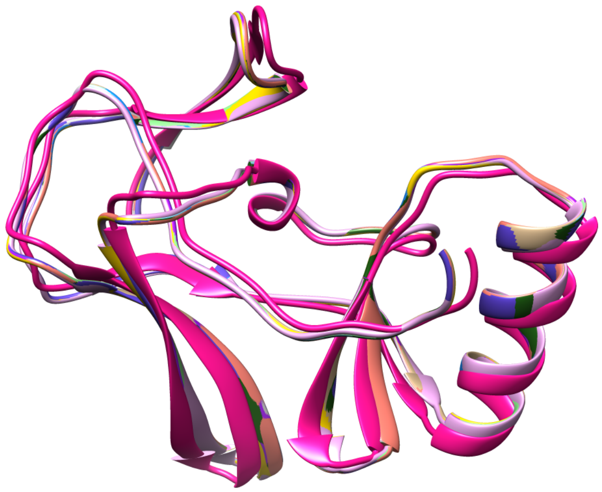

Supplementary figure 11. AWS domain protein structure alignment.

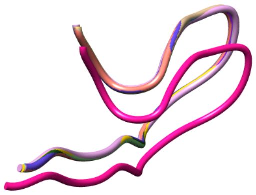

Supplementary figure 12. SET domain protein structure alignment.

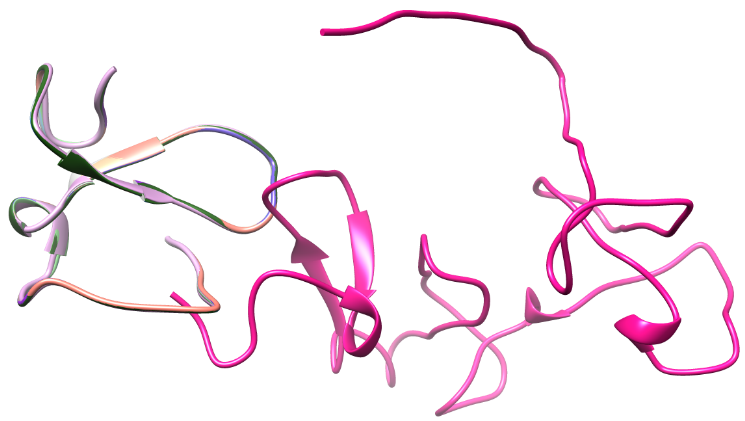

Supplementary figure 13. Post-SET domain protein structure alignment.

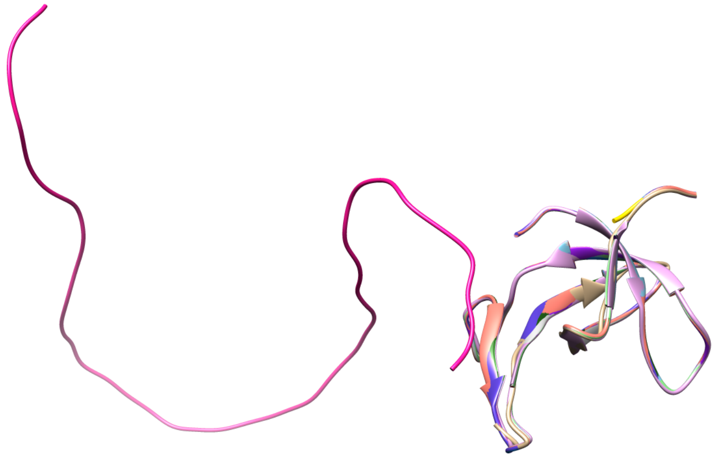

Supplementary figure 14. Localization of SD1 and SD2 on the modelled structure. Both structures were divided taking in count the spacial and functional distribution of the regions in sequence and space. In purple SD1, formed by PWWP1; in orange SD2, formed by PWWP2, 1-4PHD, RING, AWS, SET and 1Post-SET domains.

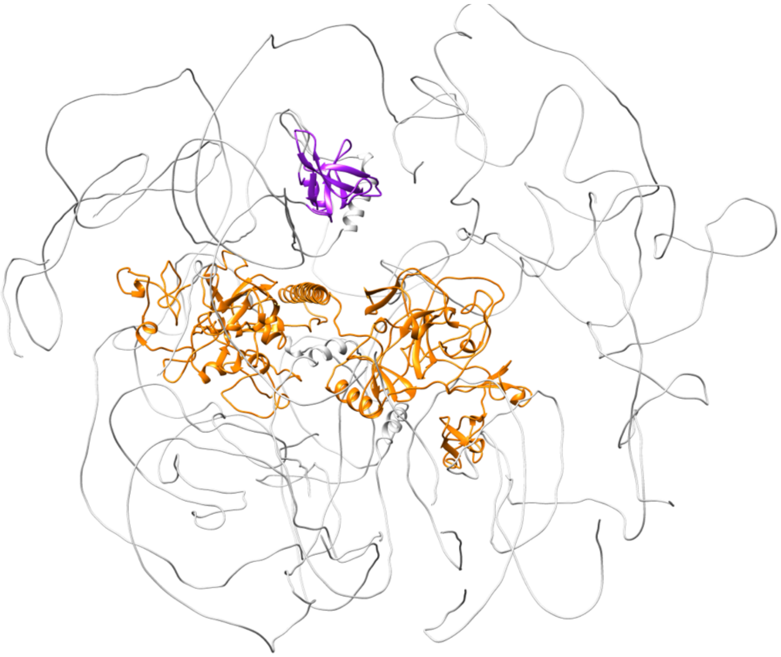

Supplementary figure 15. SD2 distribution of functional domains. Super domain 2 was divided in two for a more comprehensive distribution analysis. a) SD2 SET catalytic domain region, all core catalytic and accessory components for the methylation of lysine 9, lies between this neighborhood, as well as the control region known as INHLOOP (inhibitory loop). Blue: AWS, yellow: SET, cyan: INHLOOP, purple: Post-SET. b) SD2 accessories domains known as function as secondary accessory regions for a correct function in signaling and anchorage for the rest of the catalytic complex. Yellow: PHD1, purple: PHD2, cyan: RING, blue: PHD3, green: PHD4, red: PWWP2.

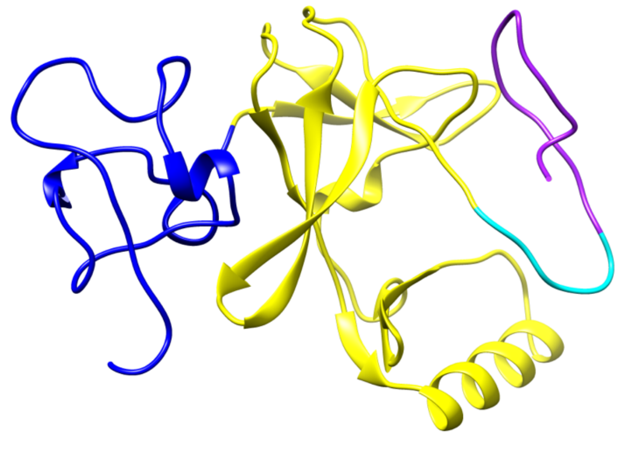

a)

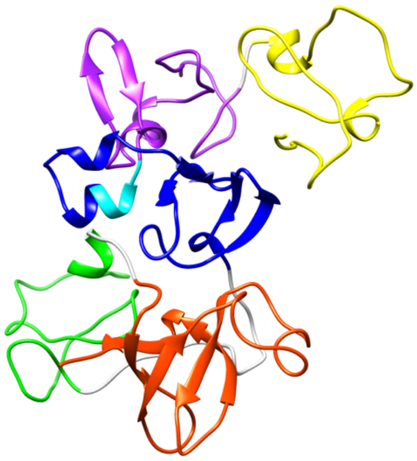

b)
